# Social Determinants of Alzheimer’s Disease and Related Dementias: Racial and Regional Variations

**DOI:** 10.1093/geroni/igab046.382

**Published:** 2021-12-17

**Authors:** Roy Martin, Liang Shan, David Geldmacher, Giovanna Pilonieta, Richard Kennedy, Gabriela Oates, Maria Pisu

**Affiliations:** 1 University of Alabama at Birmingham, Birmingham, Alabama, United States; 2 University of Alabama at Birmingham, BIrmingham, Alabama, United States; 3 University of Alabama at Birmingham, University of Alabama at Birmingham, Alabama, United States; 4 University of Alabama At Birmingham, Birmingham, Alabama, United States

## Abstract

To examine whether racial and regional social determinants of health disparities exist for older adults with Alzheimer’s disease and related dementias (ADRD). We identified 115,879 African American (AA) and White older adults with ADRD (10% from the Deep South) from administrative claims data for a 5% random sample of Medicare beneficiaries (2013-2015). We examined racial and regional differences across sociodemographic characteristics, county-level linked poverty indicators, medical resource availability categories, insurances quality indicators. Social context differences were found between regions including Deep South older adults with ADRD having higher economic impoverishment and lower access rates to specialty medical care services. Older Deep South AA had higher Medicare/Medicaid eligibility rates and less medical access. Significant socioeconomic disparities exist between Deep South and other US regions across several social determinant factors in older adults with ADRD. Social context differences were especially prominent for older Deep South AA with ADRD.

